# A Novel Device for Smartphone-Based Fundus Imaging and Documentation in Clinical Practice: Comparative Image Analysis Study

**DOI:** 10.2196/17480

**Published:** 2020-07-29

**Authors:** Maximilian W M Wintergerst, Linus G Jansen, Frank G Holz, Robert P Finger

**Affiliations:** 1 Department of Ophthalmology University Hospital Bonn Bonn Germany

**Keywords:** smartphone-based fundus imaging, smartphone-based funduscopy, smartphone, retinal imaging, mHealth, mobile phone, smartphone imaging, smartphone funduscopy, smartphone ophthalmoscope

## Abstract

**Background:**

Smartphone-based fundus imaging allows for mobile and inexpensive fundus examination with the potential to revolutionize eye care, particularly in lower-resource settings. However, most smartphone-based fundus imaging adapters convey image quality not comparable to conventional fundus imaging.

**Objective:**

The purpose of this study was to evaluate a novel smartphone-based fundus imaging device for documentation of a variety of retinal/vitreous pathologies in a patient sample with wide refraction and age ranges.

**Methods:**

Participants’ eyes were dilated and imaged with the iC2 funduscope (HEINE Optotechnik) using an Apple iPhone 6 in single-image acquisition (image resolution of 2448 × 3264 pixels) or video mode (1248 × 1664 pixels) and a subgroup of participants was also examined by conventional fundus imaging (Zeiss VISUCAM 500). Smartphone-based image quality was compared to conventional fundus imaging in terms of sharpness (focus), reflex artifacts, contrast, and illumination on semiquantitative scales.

**Results:**

A total of 47 eyes from 32 participants (age: mean 62.3, SD 19.8 years; range 7-93; spherical equivalent: mean –0.78, SD 3.21 D; range: –7.88 to +7.0 D) were included in the study. Mean (SD) visual acuity (logMAR) was 0.48 (0.66; range 0-2.3); 30% (14/47) of the eyes were pseudophakic. Image quality was sufficient in all eyes irrespective of refraction. Images acquired with conventional fundus imaging were sharper and had less reflex artifacts, and there was no significant difference in contrast and illumination (*P*<.001, *P*=.03, and *P*=.10, respectively). When comparing image quality at the posterior pole, the mid periphery, and the far periphery, glare increased as images were acquired from a more peripheral part of the retina. Reflex artifacts were more frequent in pseudophakic eyes. Image acquisition was also possible in children. Documentation of deep optic nerve cups in video mode conveyed a mock 3D impression.

**Conclusions:**

Image quality of conventional fundus imaging was superior to that of smartphone-based fundus imaging, although this novel smartphone-based fundus imaging device achieved image quality high enough to document various fundus pathologies including only subtle findings. High-quality smartphone-based fundus imaging might represent a mobile alternative for fundus documentation in clinical practice.

## Introduction

Imaging of the eye using smartphones has become increasingly popular and allows for an inexpensive and mobile fundus examination and documentation [1,2]. Although initially the lens needed to be held in the optical path manually [1-3], different proprietary adapters are available now [4-7]. Smartphone-based fundus imaging can be performed based on both direct [4,5] and indirect ophthalmoscopy [6,8-10]. Given the low costs and great mobility, smartphone-based fundus imaging has the potential to revolutionize eye care, especially in lower-resource settings [11,12]. Furthermore, smartphones allow for continuous connectivity, and hence, smartphone-based fundus imaging might pave the way for telemedicine in ophthalmology [13].

With the advent of smartphone-based fundus imaging, multiple applications in ophthalmology including smartphone-based diabetic retinopathy screening [14-20] and smartphone-based optic nerve head evaluation [5,21,22] have become available. In addition, smartphone-based fundus imaging has successfully been used for documentation of hypertensive retinopathy [23] and its utility for fundus documentation in a pediatric emergency department has been evaluated [24]. Furthermore, direct smartphone-based fundus imaging might have merits in teaching medical students and other health care trainees fundus examination as it seems much easier to understand and master than conventional direct funduscopy [25,26].

Here, we evaluated a novel, high-quality optics adapter for smartphone-based fundus imaging in single-image acquisition or video mode for documentation of a variety of retinal/vitreous pathologies in a patient sample with wide refraction and age ranges.

## Methods

### Subject Recruitment

Patients were consecutively recruited from the retina outpatient clinic at the Department of Ophthalmology of the University of Bonn, Germany. Ethical approval was obtained from the Ethics Committee of the University of Bonn (approval ID 209/16), and informed consent was obtained from all study participants or their legal guardians prior to study initiation. The Declaration of Helsinki was followed. Exclusion criteria were severe media opacities or any contraindications for pupil dilation.

### Image Acquisition

**Figure 1:**
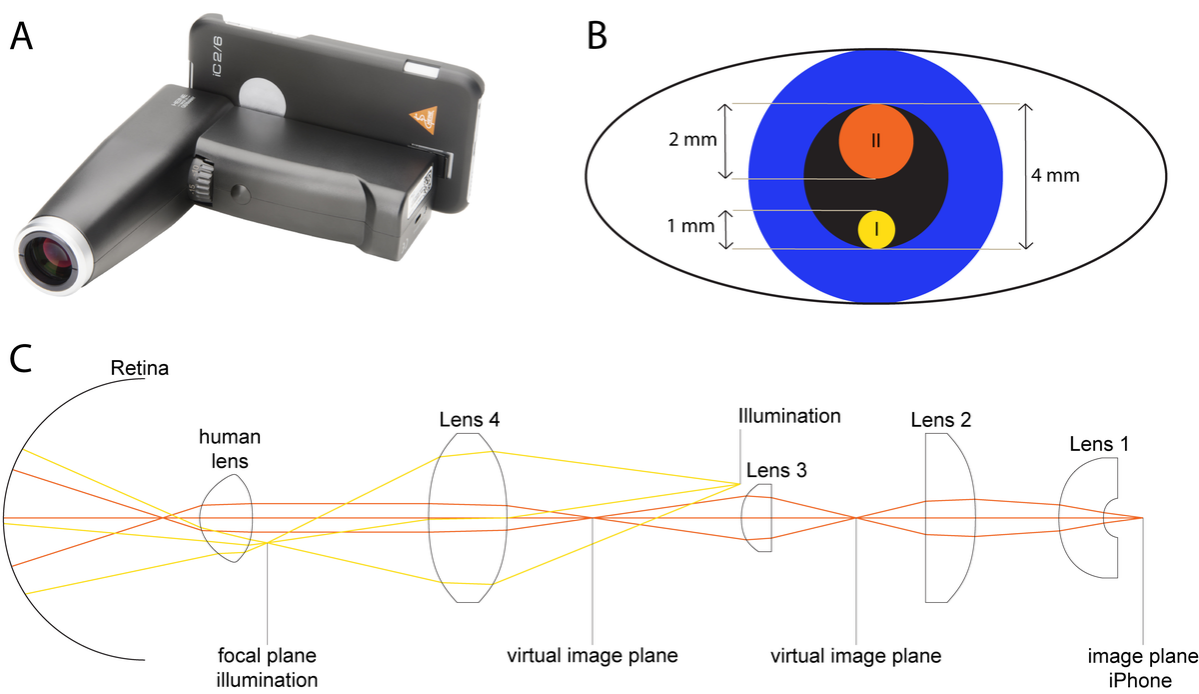
Optical setup of the smartphone-based funduscopy device evaluated in this study. The (A) smartphone-based fundus imaging device (iC2 funduscope; HEINE Optotechnik GmbH & Co. KG) evaluated in this study has a separate optical path for the illumination system and the imaging system. (B) Optical paths in the plane of the pupil (I=illumination’s light path, II=imaging system’s light path) with approximate scale. (C) The optical setup consists of four aspheric lenses and a polarized illumination source with a 3000-K light-emitting diode. The device allows for a field-of-view of up to 34 °. Lens 3 is adjustable for compensations of refractions up to ±15 D.

Eyes were dilated with tropicamide (5.0 mg/mL) and phenylephrine (100 mg/mL) and imaged with the iC2 funduscope (HEINE Optotechnik GmbH & Co. KG; Figure 1) using an iPhone 6 (Apple Inc.). The smartphone-based fundus imaging device allows for a retinal field of view of up to 34 °, has a CE sign, and is classified as an ophthalmologic instrument group 1 according to the international DIN EN ISO 15004-2 standard. The weight of this handheld smartphone-based fundus imaging device is 300 g and it can be operated by one hand (focus adjustment and trigger for image acquisition), while the other hand placed on the forehead of the patient can be used to stabilize the device. Focus can be adjusted via a 2-step system: (1) a wheel at the front of the casing (Figure 1A) allows for a first manual compensation of the approximate refraction (±15 D); and (2) the autofocus of the smartphone camera achieves a fine adjustment of the focus (±3 D) after slightly pushing the image-acquisition trigger below the refraction wheel to its first stage. The image is then acquired by pushing the trigger to its second stage. Supported smartphones include the iPhone 8, 7, 6, 6s, 5s, and 5se. Images can be exported from the smartphone for further use. The app allows for a quick examination for immediate imaging without the need of entering patient data beforehand. Fundus illumination is adjustable via three brightness levels (50%, 75%, and 100% illumination). The device comes with an iOS app and the iPhone and smartphone-based fundus imaging device are connected via Bluetooth. Smartphone-based fundus imaging was performed by the same examiner (MWMW) in all patients in a darkened room. No scleral indentation was used for peripheral examination. Smartphone-based fundus imaging was performed in single-image acquisition (round images, image resolution of 2448 × 3264 pixels) or video mode (slightly narrowed images, image resolution of 1248 × 1664 pixels). In addition, a subgroup of the eyes were dilated and imaged with conventional fundus imaging (VISUCAM 500; Carl Zeiss Meditec). In case fundus images were optimized for brightness, the postprocessing was equally performed in the whole image, with no selective adjustments in parts of the image made.

### Image Analysis

Images were qualitatively analyzed for sharpness as previously reported [22]; reflex artifacts (on a scale from 0 to 3; 3=reflex artifacts in <10% of the field of view; 2=reflex artifacts in >10% of the field of view; 1=reflex artifacts in >30% of the field of view; and 0=reflex artifacts in >50% of the field of view); and contrast and illumination (on a scale from 1 to 3; 3=all vessels contrast well against fundus pigmentation; 2=most vessels contrast against fundus pigmentation; and 1=only few retinal vessels visible). Usable field of view, which was not blocked by image artifacts, was assessed. The Kruskal–Wallis test was used for multiple comparison between groups for nonparametric data.

## Results

**Figure 2:**
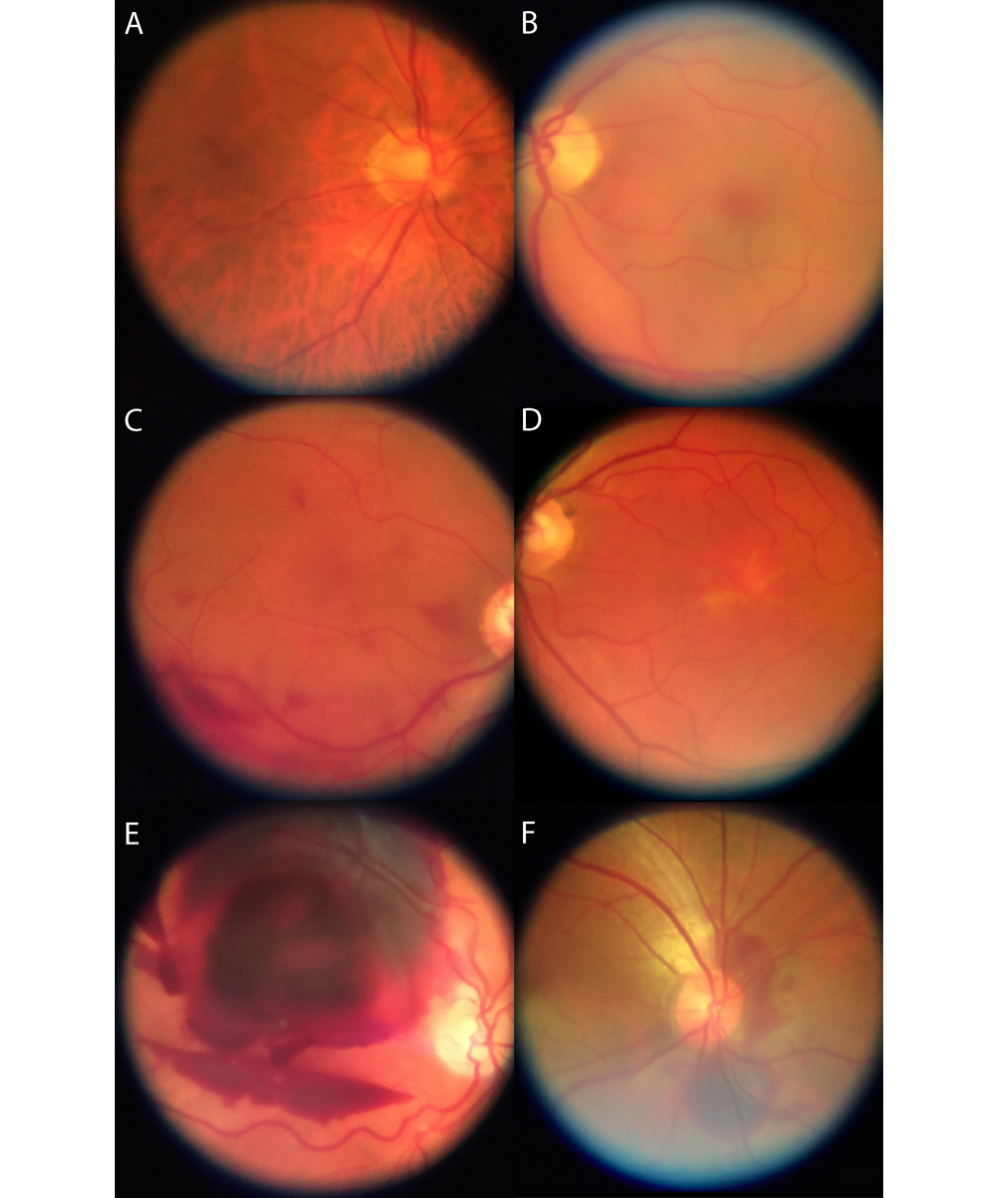
Exemplary smartphone-based fundus images of the posterior pole. (A) Healthy eye with slight peripapillary atrophy, (B) central retinal artery occlusion with a cherry red spot, (C) multifocal preretinal subhyaloidal hemorrhage, (D) adult vitelliform macular dystrophy, (E) massive subretinal combined with subhyaloidal hemorrhage, and (F) traumatic peripapillary choroidal rupture.

**Figure 3:**
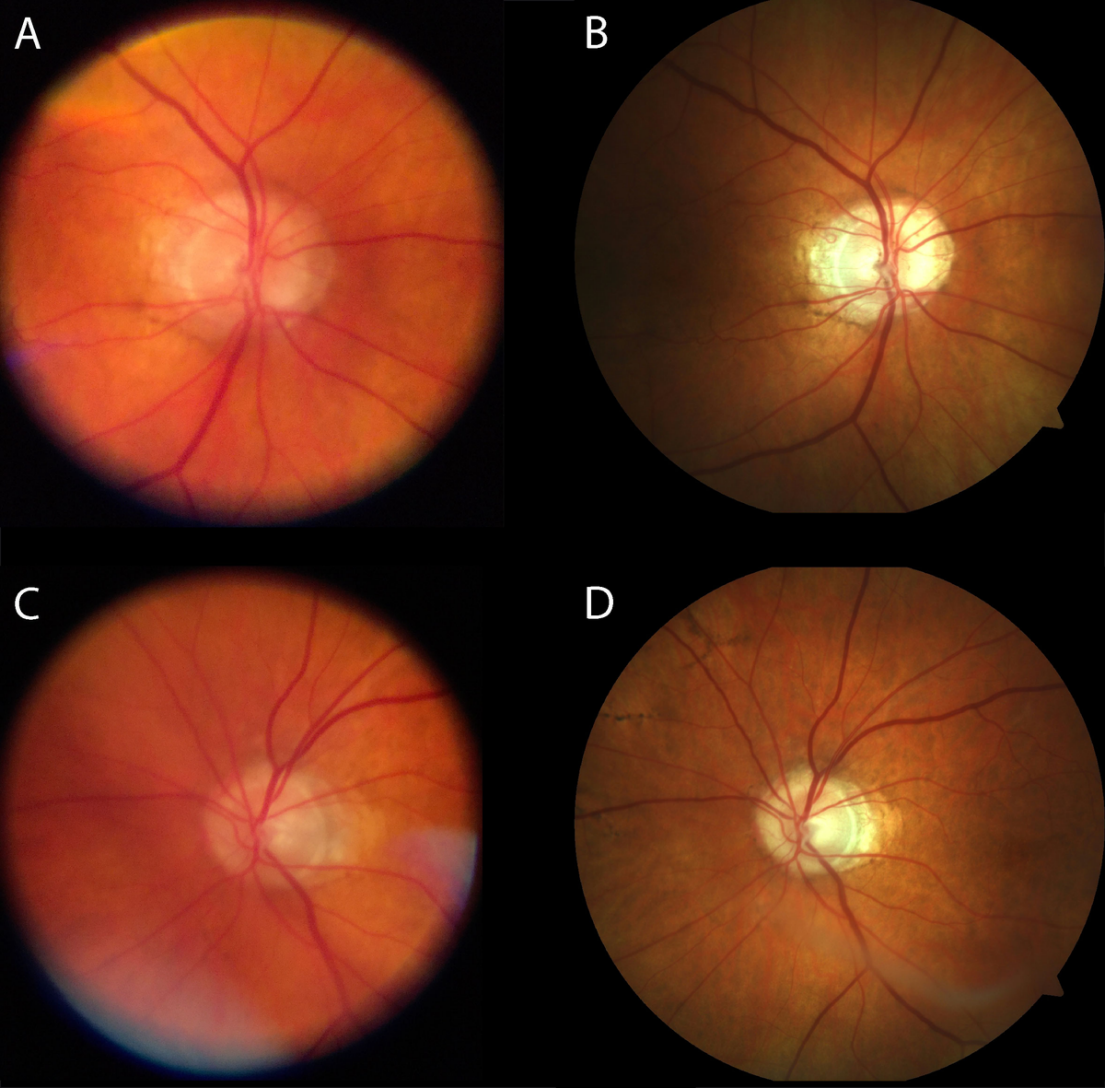
Exemplary comparison of smartphone-based fundus imaging and conventional fundus imaging. Exemplary comparison of two eyes (A+B and C+D) which were both imaged with smartphone-based fundus imaging (A and C) and conventional fundus imaging (B and D).

**Figure 4:**
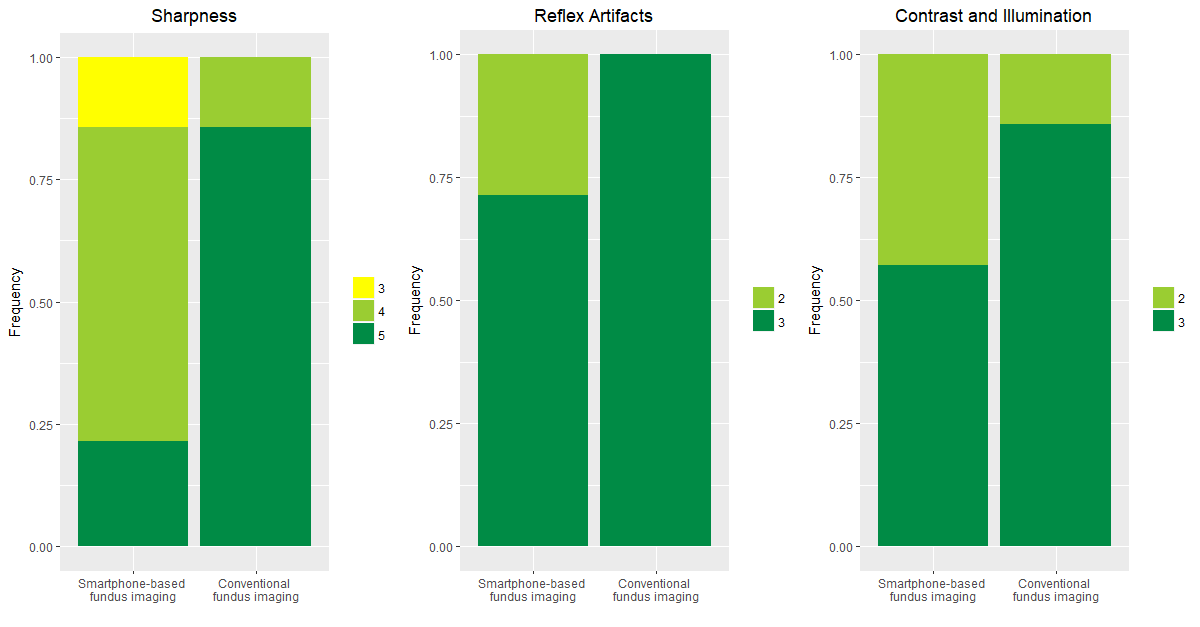
Comparison of image quality between smartphone and conventional fundus imaging. Image quality in terms of sharpness (left; semiquantitative scale from 1 to 5 as previously reported [22]), reflex artifacts (middle; scale from 0 to 3; 3=reflex artifacts in <10% of the field of view; 2=reflex artifacts in >10% of the field of view; 1=reflex artifacts in >30% of the field of view; 0=reflex artifacts in >50% of the field of view), and contrast and illumination (right; scale from 1 to 3; 3=all vessels contrast well against fundus pigmentation; 2=most vessels contrast against fundus pigmentation; 1=only few retinal vessels visible) is shown for smartphone-based fundus imaging and conventional fundus imaging.

A total of 47 eyes of 32 participants with healthy eyes or the following vitreous/retinal disease were recruited (see Table 1 for characteristics of the sample): healthy (8 eyes), exudative age-related macular degeneration (6 eyes), retinal detachment (4 eyes), adult-onset vitelliform macular dystrophy (4 eyes), intermediate-stage age-related macular degeneration (3 eyes), status post retinal surgery (3 eyes), dome-shaped maculopathy (2 eyes), choroidal nevus (2 eyes), uveal coloboma (2 eyes), central serous chorioretinopathy (2 eyes), central retinal artery occlusion (1 eye), retrohyaloidal hemorrhage (1 eye), Coats disease (1 eye), status post blunt ocular trauma with choroidal rupture (1 eye), posterior uveitis (1 eye), branch retinal vein occlusion (1 eye), retinoschisis with retinal detachment (1 eyes), status post retinal gunshot injury (1 eye), asteroid hyalosis (1 eye), peripheral retinal hole (1 eye), and endogenous endophthalmitis (1 eye). Visualization of the fundus using smartphone-based fundus imaging was possible in all eyes following dilation, irrespective of refraction. Image quality was sufficient to document various specific fundus pathologies including pathologies with only subtle findings as in macular dystrophy, as demonstrated in the exemplary images of the posterior pole in Figure 2. A total of 14 eyes were imaged by both smartphone-based fundus imaging and conventional fundus imaging (see Figure 3 for exemplary images). Images acquired with conventional fundus imaging were sharper and had less reflex artifacts, compared with images acquired by smartphone-based fundus imaging (*P*<.001 and *P*=.03; Figure 4). There was no significant difference in contrast and illumination (*P*=.10; Figure 4), and usable field of view was not different between smartphone-based fundus imaging and conventional fundus imaging—0.99% (0.03%) and 100% (0%), respectively (*P*=.07).

**Figure 5:**
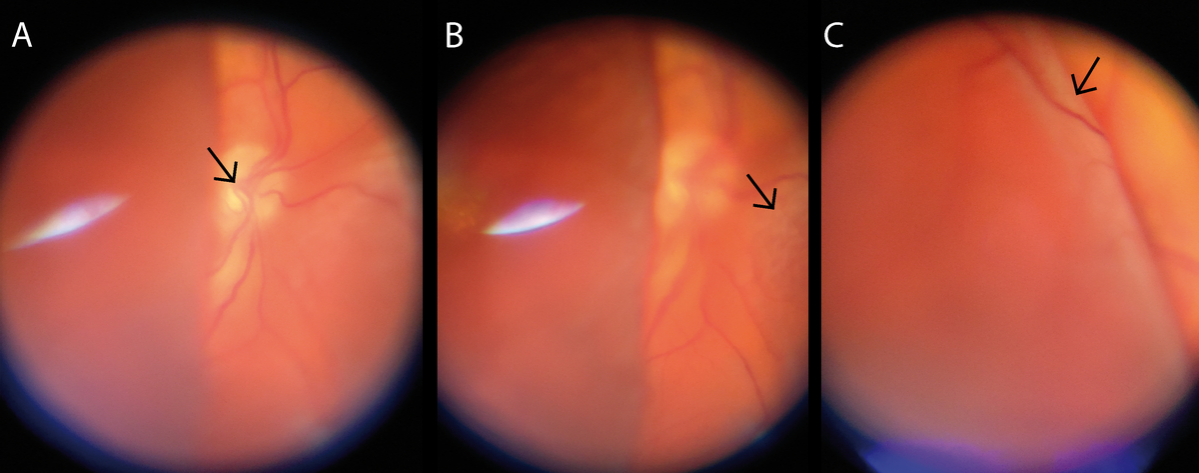
Documentation of different focal planes with smartphone-based fundus imaging in a total retinal detachment. With varying focus, different focal planes (indicated by black arrows) from (A) the optic disc to (B) nasally detached retina to (C) temporal bulla of detached retina can be documented. Note the characteristic reflex artifact in (A) and (B) which can predominantly be seen in pseudophakic eyes.

**Figure 6:**
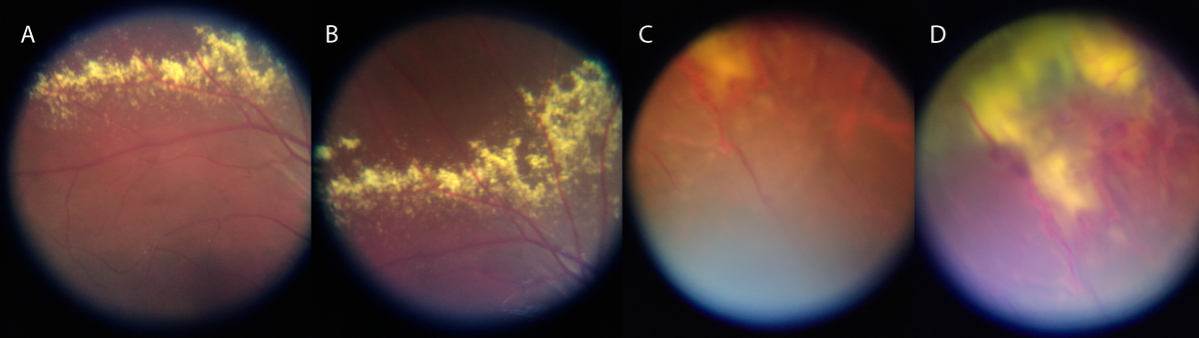
Smartphone-based fundus images from a 7-year-old child with Coats disease. (A) and (B) exudates superior from the upper arcade; (C) and (D) massive exudation and telangiectasia of retinal vessels in the far superior-temporal periphery, which were also funduscopically difficult to see.

**Figure 7:**
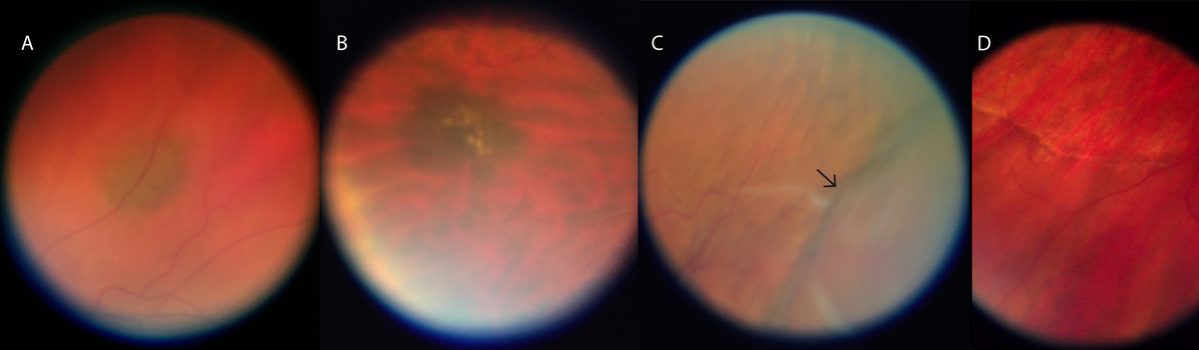
Exemplary smartphone-based fundus images from the mid periphery. (A) and (B) Choroidal nevi, (C) choroidal detachment (the out-of-focus area indicated by the black arrow), and (D) a subretinal demarcation line (high water mark) in a case of longstanding retinal detachment.

Adjustable depth of focus allowed for documentation of retinal detachments in varying focal planes (Figure 5). Applicability in children was demonstrated by imaging a 7-year-old child with Coats disease (Figure 6). We found image quality to be limited in the far periphery, but sufficient to detect the Coats-specific pathological alterations (Figures 6C and 6D). In contrast to the far periphery, the midperiphery could be imaged with only slightly reduced image quality as compared with the posterior pole (Figure 7).

Documentation of pathology was more easily achieved using the video mode than the single-image acquisition mode. Multimedia Appendix 1 shows an exemplary smartphone-based fundus examination in video mode where first the healthy right eye and then the left eye exhibiting vitreous synchysis were imaged. Furthermore, smartphone-based fundus imaging in video mode with slightly altering camera directions conveys a 3D impression of structures such as deepened optic nerve cups (Multimedia Appendix 2). We termed this mock 3D impression *pseudo-biomicroscopic effect.*

**Figure 8:**
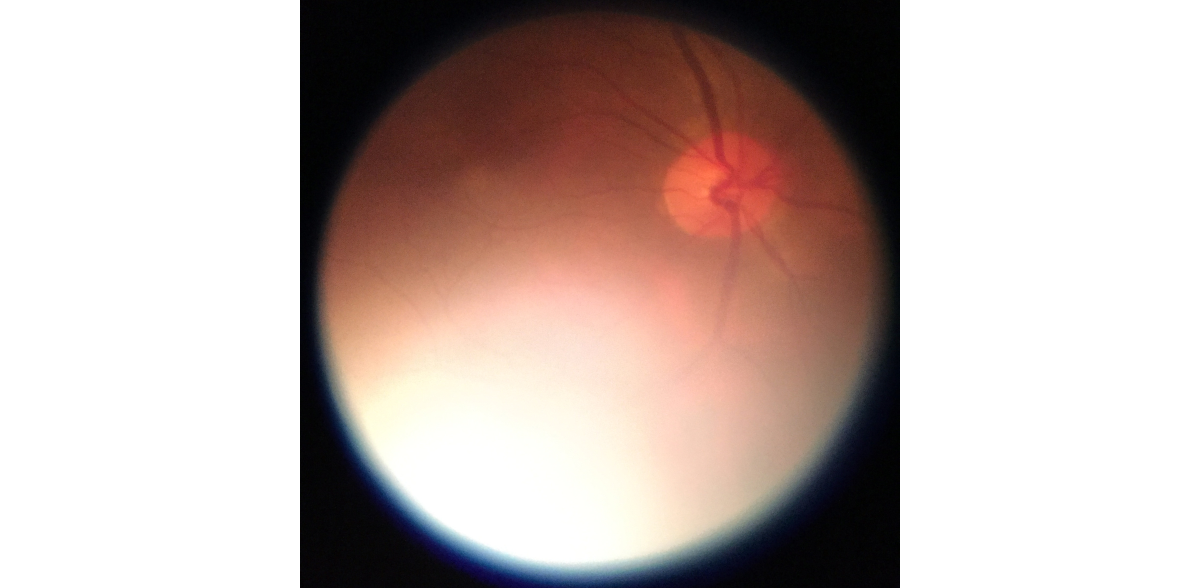
Smartphone-based fundus image of the posterior pole acquired without pupil dilation. Compared to imaging with pupil dilation, field of view is constrained due to increased glare from the image periphery.

Glare of the image periphery (Figures 2F and 7B) and minor reflexes (Figures 5A and 5B) were image artifacts that we encountered during both single-image acquisition and video mode, with the latter artifact being predominantly present in pseudophakic eyes. Glare increased as images were acquired from more peripheral parts of the retina (Figures 6C and 6D). Smartphone-based fundus imaging with this device was also possible without pupil dilation but we found it to be more difficult and time consuming (>2 minutes for one image of the posterior pole); furthermore, reduced field of view due to increased glare limits its application (see Figure 8 for an exemplary image acquired without pupil dilation).

**Table 1:** Characteristics of the study sample (N=47).

Characteristic		Value
Age (years), mean (SD); range		62.3 (19.8); 7 to 93
**Sex, n (%)**		
	Male	29 (62)
	Female	18 (38)
**Lens status, n (%)**		
	Phakic, clear lens	33 (70)
	Pseudophakic	14 (30)
Visual acuity (logMAR), mean (SD); range		0.48 (0.66); 0 to 2.3
Refraction (spherical equivalent; D), mean (SD); range		–0.78 (3.21); –7.88 to 7.0

## Discussion

Smartphone-based fundus imaging with this device allows for retinal imaging of a diverse group of retinal pathologies in adults and children. Best image quality is achieved at the posterior pole and decreases with more peripheral imaging. Use of the video mode had several advantages over single-image acquisition mode but comes with a higher data volume, reduced image resolution, and the need for postprocessing (ie, selection of relevant parts of the video).

Our study further supports existing data showing that retinal imaging using smartphone-based fundus imaging can be successfully applied to a variety of ophthalmologic diseases and might be implemented in health care systems [5,14-19,21-24]. Smartphone-based fundus imaging might be especially applicable in children as they are often familiar with and interested in smartphones. For example, it has been shown that smartphone-based fundus imaging can be used in a pediatric emergency department setting for screening for fundus pathologies [24].

Image quality of conventional fundus imaging was superior to that of smartphone-based fundus imaging, yet smartphone-based image quality was sufficient to document various fundus pathologies, including only subtle findings. Imaging the peripheral retina is possible; however, due to optical limitations—also related to the eye not being a perfect optical system [27]—image quality is reduced. Glare increased with more peripheral imaging and was particularly pronounced without pupil dilation. This might indicate a correlation of image glare with the effective aperture in the optical path. As the viewing angle increases in imaging of the mid and especially far retinal periphery, the effective aperture at the level of the pupil gets smaller and exhibits asymmetric distortions [28]; therefore, among other imaging artifacts, light scatter increases, which leads to increased glare in the image periphery. In addition, we observed characteristic small reflex artifacts which were predominantly seen in pseudophakic eyes, albeit not reducing overall image quality. Cataract might be a further confounder of image quality in smartphone-based fundus imaging; however, there were no major lens opacities in our study population.

Smartphone-based fundus imaging without pupil dilation might appear promising as no topical application of drugs is needed and examination can be performed directly without any waiting time. Although this device was not specifically built for imaging without pupil dilation, we showed that it is in principle possible, but not feasible due to reduced image quality and increased examination time. Yet, there are other approaches for smartphone-based fundus imaging which are capable of image acquisition without pupil dilation [4,24,29]. However, pupil dilation is still recommended to achieve best image quality in smartphone-based fundus imaging [22].

Smartphone-based fundus imaging using this device allows for retinal imaging of a diverse group of retinal pathologies in adults and children. Using the video mode has several advantages over single-image acquisition mode but comes with a higher data volume, reduced image resolution, and the need for postprocessing. We showed that video documentation of deepened optic disc cups conveys a mock 3D impression, which we termed *pseudo-biomicroscopic effect.* Although smartphone-based fundus imaging using video mode has already been reported [30], this is the first time this mock 3D impression is described. The pseudo-biomicroscopic effect might aid evaluation of optic nerve heads, for example, in the setting of a lower-resource glaucoma screening.

The strengths of our study are a comparison with conventional fundus imaging, semiquantitative analyses of image quality, a detailed description of the device, an application to a variety of different retinal diseases, wide refraction and age ranges of participants, and a novel approach for video-based documentation of the 3D structure of the optic nerve cup. However, we did not compare this device with other smartphone-based fundus imaging adapters, had a relatively small sample size, and did not evaluate applicability in eyes with clinically relevant lens opacities.

Image quality of conventional fundus imaging was superior to that of smartphone-based fundus imaging in terms of sharpness and reflex artifacts, although this novel smartphone-based fundus imaging device achieved image quality high enough to document various fundus pathologies, including only subtle findings. Hence, smartphone-based fundus imaging with this device might represent a mobile alternative for high-quality fundus documentation, for example, in immobilized intensive care patients. An image quality comparison between different smartphone-based fundus imaging approaches and further studies on the applicability of this device are warranted.

## Appendix

Multimedia Appendix 1 Exemplary smartphone-based fundus examination of a healthy eye and an eye with vitreous synchysis. Both eyes are from the same patient and first the healthy right eye and then the left eye exhibiting vitreous body synchysis are documented. The second eye is examined from 27 seconds onward.

Multimedia Appendix 2 Pseudo-biomicroscopic effect. An eye with a deepened optic nerve cup was examined with smartphone-based fundus imaging in video mode. Examination from slightly altered directions generates a mock 3D impression.
